# A protocol to prospectively assess risk factors for medial tibial stress syndrome in distance runners

**DOI:** 10.1186/s13102-018-0109-1

**Published:** 2018-11-22

**Authors:** Joshua Mattock, Julie R. Steele, Karen J. Mickle

**Affiliations:** 1 Biomechanics Research Laboratory, University of Wollongong, Wollongong, NSW Australia; 2 Institute of Health and Sport, Victoria University, Melbourne, VIC Australia

**Keywords:** Medial tibial stress syndrome, Injury prevention, Running injuries, Protocols

## Abstract

**Background:**

Medial tibial stress syndrome (MTSS) is a lower leg injury with a reported incidence rate of up to 35% in active individuals. Although numerous prospective studies have tried to identify risk factors for developing MTSS, managing the syndrome remains difficult. One risk factor yet to be extensively explored in MTSS development is reduced lower leg girth. Further investigation of reduced lower leg girth is required due to the important role lower leg musculature plays in attenuating ground reaction forces during the gait cycle. Therefore, the primary aim of this study is to ascertain whether lower leg muscle morphology and function contribute to the development of MTSS. Our ultimate aim is to identify potential risk factors for MTSS that can be targeted in future studies to better manage the injury or, preferably, prevent individuals developing MTSS.

**Methods:**

This study will be prospective in design and will recruit asymptomatic distance runners. All participants will be tested at base line and participants will have their training data longitudinally tracked over the following 12 months to assess any individuals who develop MTSS symptoms. At base line, outcome measures will include bilateral measures of lower limb anthropometry; cross sectional area (CSA) and thickness of the tibialis anterior, peroneals, flexor digitorum longus, flexor hallucis longus and thickness of soleus, medial and lateral head of gastrocnemius. Tibial bone speed of sound, ankle dorsiflexion range of motion, strength of the six previously described muscles, foot alignment and ankle plantar flexor endurance will also be assessed. Participants will also complete a treadmill running protocol where three-dimensional kinematics, plantar pressure distribution and electromyography data will be collected.

**Discussion:**

This study will aim to identify characteristics of individuals who develop MTSS and, in turn, identify modifiable risk factors that can be targeted to prevent individuals developing this injury.

## Background

Medial tibial stress syndrome (MTSS) is an exercise-induced injury of the posteromedial tibial border. Pain is of a diffuse nature, covering an area of at least 5 cm at the middle to distal third of the tibia [1]. Clinically, MTSS is considered a separate pathology from stress fracture, chronic exertional compartment syndrome and other neuropathies affecting the lower leg [2]. Military personal, distance runners and athletes involved in jumping sports predominately incur MTSS with a reported incidence rate of between 4 and 35% [1–4].

Recent studies suggest MTSS is most likely caused by a bone stress reaction of the tibial cortex as a result of tibial bending and subsequent bone remodelling [1, 5–8]. It is hypothesised that remodelling of the tibial cortex results in a relatively osteopenic bone [6, 8], which is unable to withstand repetitive loading experienced by individuals who continue to complete large training volumes. Bony adaptations occur predominately at the site where bending forces are greatest, coinciding with the narrowest cross sectional area of the tibia [9, 10].

In order to treat and ideally prevent development of MTSS, identifying risk factors for MTSS has featured prominently in the literature [2, 3, 11–19]. Major risk factors for MTSS are thought to include increased navicular drop, pronated foot type, increased body mass index (BMI), fewer years running experience, a history of MTSS and female gender. Despite identifying these risk factors, however, MTSS management remains difficult as many of these factors cannot be modified. In fact, the authors of a systematic review of treatment interventions for MTSS concluded that there is no high-quality evidence for the effect of any intervention [20]. As a result, further exploration of risk factors involved in MTSS development is needed upon which to base effective treatment strategies.

Although MTSS and tibial stress fractures are considered separate pathologies, they share a similar aetiology involving repetitive loading of the lower limb. For management purposes it has been proposed that MTSS and tibial stress fracture likely exist on a bone stress-failure continuum where MTSS is a relatively mild expression and stress fracture is a severe presentation [5]. Therefore, it is postulated that certain risk factors for developing stress fracture are also implicated in developing MTSS [5].

One risk factor linked to stress fracture development but yet to be extensively explored in MTSS development is reduced lower leg girth. Reduced lower leg girth is reported to influence the ability of the lower limb to attenuate ground reaction forces and, in turn, the amount of load transferred to the tibia [21]. A prospective study by Burne et al. [14] assessed risk factors associated with the development of exertional medial tibial pain (EMTP), which includes MTSS, tibial stress fracture, chronic exertional compartment syndrome and muscular and tendon injuries. The authors of this study concluded that male military recruits who had a reduced lower leg girth were at an increased risk of developing EMTP [14]. Furthermore, a prospective study of track and field athletes revealed that female athletes who had a reduced lower leg girth were at an increased risk of developing tibial stress fractures [22]. Bennell et al. [22] also reported that every 10-mm reduction in lower leg girth represented a fourfold increased risk of tibial stress fracture, possibly related to a reduced shock absorbing capacity of the muscles increasing forces on bone. Further support for reduced lower leg girth in MTSS symptomatic individuals came from a study in which 20 cases of MTSS were assessed over a 10 year period [23]. The researchers concluded that MTSS symptomatic individuals displayed atrophy at the level of maximal muscle mass of the anterior tibial muscle group and gastrocnemius on the affected side, which had an average reduction in lower leg circumference of 1.46 cm.

As well as reduced lower leg size, reduced ankle plantar flexor muscle endurance has also been identified as a risk factor for MTSS. That is, Madeley et al. [24] reported a significant difference in the number of single leg heel raises completed by MTSS symptomatic individuals (mean 23 ± S.D. 5.6) compared to asymptomatic controls (mean 33 ± S.D. 8.6).

The mechanism for reduced lower leg muscle circumference and endurance contributing to MTSS symptoms has been attributed, in part, to poor ground reaction force attenuation during running [21]. Wakeling et al. [21] hypothesised that lean muscle mass supporting the lower limb might ultimately determine its capacity to adapt positively to loading forces and withstand injury. We therefore postulate that lower leg muscle size will dictate its ability to attenuate ground reaction forces and, subsequently, the amount of tibial bending, which could ultimately lead to development of MTSS. Currently, there is a lack of literature to describe individual muscle characteristics in a MTSS symptomatic population, with previous studies basing conclusions solely upon measurements of overall lower leg circumference [14, 22, 23]. The paucity of research describing the morphology of individual lower leg muscles limits our understanding of the composition of these muscles and how they function in MTSS symptomatic individuals compared to asymptomatic controls. Therefore, further research is required to examine the role that individual lower leg muscles play in the gait cycle and how they might contribute to the development of MTSS. It also remains unclear whether lean lower leg girth is a primary cause, or an effect, of MTSS [2].

Previous prospective studies have assessed risk factors contributing to the development of MTSS in both military and running populations [2, 3, 13, 14, 17, 19]. However, these studies have limited their outcome variables to those obtained from questionnaires, simple anthropometric measures or static lower limb strength measures [2, 3, 13, 14, 17, 19]. Furthermore, application to distance running populations is limited with the studies by Sharma et al. [17], Burne et al. [14] and Yates et al. [2] being conducted in military populations and the studies by Bennett et al. [3], Hubbard et al. [19] and Plisky et al. [13] being restricted to young adult (15–26 years) cross country runners and varsity athletes.

Despite several proposed risk factors associated with MTSS development, clinically, we remain unable to provide better management options other than prolonged rest [11]. Given the similarities in the aetiological mechanism between MTSS and stress fracture development and the role the lower limb muscles plays in attenuating ground reaction forces during the stance phase of gait, there is scope to explore how lower limb morphological and functional characteristics identified in stress fracture development influence MTSS development. To date, investigations of the effects of lower leg muscle size in MTSS development have involved a gross measure of lower leg circumference at its largest girth. There is yet to be a prospective study to comprehensively assess the structure and function of the lower leg muscles in distance runners in order to identify characteristics of individuals who will develop MTSS symptoms and, in turn, identify modifiable risk factors that can be targeted to prevent MTSS development.

## Method/Design

### Aim

The primary aim of this study is to identify whether lower leg muscle morphology and function contribute to the development of MTSS. Our ultimate aim is to identify potential risk factors for MTSS that can be targeted in future studies to better manage the injury or, preferably, prevent individuals developing MTSS.

### Design

A prospective study design will be used for this study, whereby participants are assessed at baseline and then tracked for 12 months. A sample size calculation has estimated that 117 distance runners are required to provide 80% power to detect a significant difference in lower leg muscle size of 13 mm between runners who develop MTSS and those that do not (alpha set at 5%). In 2016, 30,000 runners completed either a marathon or half marathon in New South Wales, Australia [M. Grech, personal communication, May 20, 2017]. Prior studies suggest a MTSS prevalence rate of between 4 and 35% [1, 2, 4]. However, based on previous prospective studies assessing MTSS in running cohorts, we expect the prevalence rate of MTSS development over a 12-month period to be approximately 15% [3, 13, 19]. A 14.4% drop-out rate has been included in the calculations based on a previous prospective study investigating risk factors associated with stress fracture development in track and field athletes over a 12 month period [22].

**Fig. 1:**
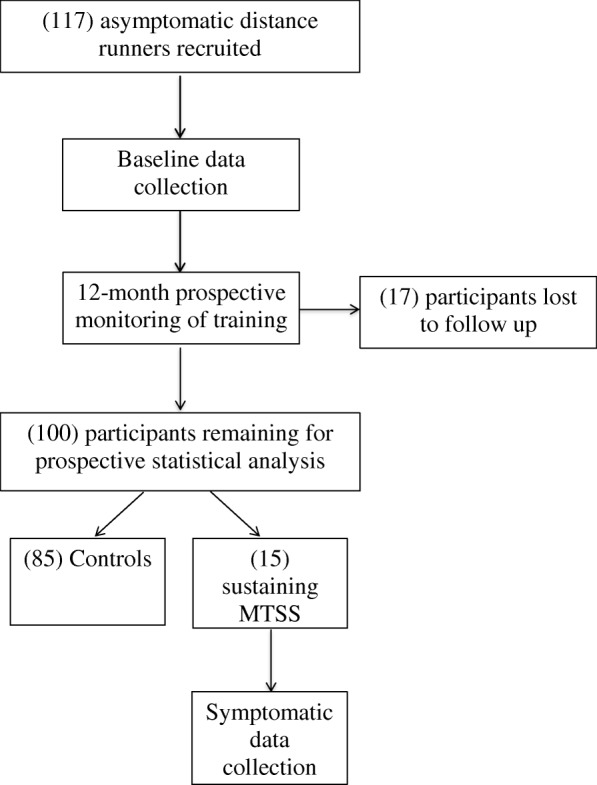
Flow chart of participant movement through study. Estimated numbers are included

All testing will be conducted at the University of Wollongong Biomechanics Research Laboratory. Runners will be recruited through social media, running and triathlon clubs and running and triathlon events throughout the Illawarra and Southern Sydney region. Participants will undergo base line testing, as described below, and then be longitudinally tracked over a 12-month period to determine the number of individuals who develop MTSS. Those participants who develop MTSS will be retested to determine changes to outcome variables from baseline assessment. The flow of participants through the study is shown in Fig. 1. The end point for each participant will either be 12-months of injury free running, the development of MTSS, or any injury that limits further participation in the study.

### Participants

Participant inclusion criteria will be male and female distance runners who have run an average of 30 km per week or more, for no less than 6 months or are in training for a long distance event of at least a half marathon (21.1 km), are aged over 18 years and possess the ability to complete the 5-min running protocol. Long distance runners were chosen as the participant cohort because they have previously been shown to be predisposed to developing MTSS [1, 25].

Participants will be excluded from the study if they have undergone major surgery on either lower limb that affects their gait or if they have experienced any musculoskeletal pathology affecting their lower limbs during the past 6 months [24]. Pathology will be defined as injury management involving rest for greater than 7 days [26]. Individuals who have begun wearing, or changed, their prescription of orthotics during the last 3 months will also be excluded due to the influence on gait caused by adapting to orthotic use [27].

### Testing procedure

Prior to testing, participants will complete a questionnaire detailing their training and medical history. Footwear and, if applicable orthotics, will be assessed by the chief investigator (JM), who is a qualified and practising podiatrist. Participants will then be assessed for the following outcome variables that characterise the morphology and function of their lower legs: basic anthropometric measurements, lower leg muscle thickness and cross sectional area (CSA), tibial bone speed of sound, ankle dorsiflexion range of motion, foot alignment, lower leg muscle strength and ankle plantar flexor endurance. The biomechanics of each participant’s gait while running on a treadmill (three-dimensional kinematics, electromyography, and plantar pressure distribution), will then be assessed to determine functional characteristics of each individual’s lower limb.

### Questionnaire

Participants will complete a questionnaire to provide information pertaining to their lifetime athletic injury history, medical history and training regime. Injury and training history will be detailed as previous research has reported a reduction in athletic exposure and history of MTSS is linked to an increased risk of MTSS development [19]. Medical history will provide information regarding factors that could affect bone health. Female runners with abnormal menses, defined as missing more than three consecutive monthly periods during the last 12 months, will be asked to note this in the questionnaire to account for the likelihood of MTSS being related to reduced bone density rather than factors associated with running [28].

### Footwear and orthotics

Participants will be asked to bring to the testing session the shoes that they complete the largest number of kilometres per week in. This footwear will be assessed for make, model and wear patterns. Individuals who wear orthotics will be have their orthotics examined, recording the type of orthotic, material, reason for prescription and duration of orthotic use**.** Footwear and orthotics will be assessed to describe their influence on lower limb kinematics.

### Anthropometric measures

Each participant’s body height; body mass; length of the leg, lower leg and foot; girth of the thigh and lower leg; and width of the knee, ankle, heel and forefoot will be measured bilaterally using a stadiometer (Holtain Ltd., Crymych, Dyfed, UK), anthropometer (Holtain Ltd., Crymych, Dyfed, UK), tape measure (Muratec-KDS, Minami-Ku, Kyoto, Japan) and calibrated scales (A&D FG-KBM. Adelaide, Australia) following International Standards for Anthropometric Assessment guidelines [29]. Each measurement will be taken three times, with an average value calculated. An assessment of limb dominance will also be undertaken by observing which foot participants lead with when stepping down off a box [30] to characterise the preferred weight acceptance limb. Girth of the thigh and lower leg will later be normalised to length of the leg and lower leg, respectively.

### Lower leg muscle morphology

Thickness and CSA of six lower leg muscles will be collected using a portable Sonosite Edge HD2 (SonoSite, Inc., Bothell, WA, USA) ultrasound machine with a 15–6 Hz linear transducer (maximum depth 6 cm). Data will be collected bilaterally for tibialis anterior (TA), the peroneals (P), soleus (SOL), flexor digitorum longus (FDL), flexor hallucis longus (FHL), and medial (GM) and lateral (GL) gastrocnemius. Muscle thickness and CSA will be measured for TA, P, FDL and FHL, whereas muscle thickness alone will be measured for the SOL, GM and GL due to constraints experienced because the width of the ultrasound probe is smaller than the CSA of these muscles. The protocol described by Crofts et al. [31] for static ultrasound imaging of FDL, P and TA will be followed, whereas SOL, GM and GL will be imaged following the protocol described by Weiss et al. [32]. Imaging of TA, FDL and P will be conducted with participants in a supine position and imaging of SOL, GM and GL will be conducted with participants in a prone position. One trained operator (JM) will conduct all imaging, with each muscle image being captured three times for later analysis. Image J (National Institute for Health, Bethesda, MD, USA) software will be used to measure CSA and muscle thickness of the previously described muscles as it has been shown to have excellent inter-rater reliability [33]. A mean of three measurements will be calculated for each muscle and condition [34].

### Tibial bone speed of sound

Tibial bone speed of sound will be assessed bilaterally using a Mini-Omni Ultrasound Bone Sonometer (Sunlight BeamMed Ltd., Israel) to describe tibial bone strength. A study assessing bone mineral density in chronic MTSS sufferers reported lower regional bone mineral density in the affected tibia of the patients compared to controls [6]. The study also reported that bone mineral density was decreased on the unaffected side in individuals with unilateral symptoms, although whether this is a cause or effect of MTSS remains unclear [6]. Several prospective studies have confirmed that tibial speed of sound measurements provide comparable evidence for classifying fracture risk to bone mineral density measurements in women but without the individuals experiencing ionizing radiation [35–37]. Tibial bone speed of sound will be assessed following the protocol described in the ultrasound operating manual (Sunlight BeamMed Ltd., Israel). In brief, the participant’s test leg will be extended and supported at the ankle while they are seated. The ultrasound transducer (1.25 MHz) will be placed at 50% of the length of the tibia and repeatedly moved from the medial aspect of the tibia to the tibial crest and back again until a speed of sound measure can be calculated by the software. At least three measurements will be recorded per participant. T- and Z-scores will then be calculated, based on speed of sound measures, using the Sunlight Mini-Omni software.

### Range of motion assessment

Range of motion (ROM) will be assessed bilaterally for the participants’ ankle and hip joints. Three measurements will be collected for both ankle and hip joint ROM with the maximum angle (degrees) recorded for each limb. Ankle dorsiflexion ROM will be assessed using the knee-to-wall test and an inclinometer (Isomed Inc., Portland, OR, USA) as described by Bennell et al. [38]. The inclinometer will be placed on the anterior tibia 15 cm distal to the tibial tuberosity to assess the number of degrees between the anterior tibia and vertical for each limb. Although current research has failed to identify reduced ankle dorsiflexion ROM as a risk factor for MTSS development [2, 15, 19], ankle dorsiflexion ROM will help describe ankle and knee joint kinematics during gait [39].

The maximum number of degrees of hip internal and external ROM will be assessed using a goniometer with participants supine and the knee and hip flexed to 90° (Gollehon, Lafayette Inc., IN, USA) as described by Burne et al. [14]. Internal and external hip ROM has previously been linked with MTSS development. Burne et al. [14] reported increased internal and external hip ROM was associated with exercise related lower leg pain, whereas more recently Yagi et al. [15] reported increased internal hip ROM increased the risk of MTSS injury. However, Moen et al. [16] contradicted these findings by reporting that reduced internal hip ROM was associated with an increased risk of MTSS development.

### Muscle strength

Muscle strength will be collected bilaterally for the TA, P, SOL, GM and GL, FDL and FHL by having each participant perform a series of 3–5 s maximal efforts against a hand held dynamometer (Gollehon, Lafayette Inc., IN, USA) and following the procedures described in Table 1. Participants will be allowed adequate familiarisation with the MVC technique before three trials for each muscle are collected [40]. Participants will rest for 30 s between trials to limit the effects of fatigue [41].

**Table 1:** Description of the participant position and action required when testing muscular strength [40]

Muscle	Participant position	Action
TA	Sitting with knee flexed	Dorsiflexion of the ankle joint and inversion of the foot without extension of the great toe.
P	Side lying	Eversion of the foot with plantar flexion of the ankle joint while applying pressure against the lateral border and sole of the foot, in the direction of inversion of the foot and dorsiflexion of the ankle joint.
SOL	Prone with knee flexed to 90°	Plantar flexion of the ankle joint, without inversion or eversion of the foot.
GM and GL	Prone with knee extended	Plantar flexion of the foot with emphasis on pulling the heel upward more than pushing the forefoot downward. For maximum pressure in this position it is necessary to apply pressure against the forefoot, as well as against the calcaneus.
FDL	Supine	Plantar flexion of the lesser digits without plantar flexion of the ankle joint.
FHL	Supine	Plantar flexion of the hallux without plantar flexion of the ankle joint or lesser digits.

### Foot posture

A static assessment of foot posture will be completed using the six point Foot Posture Index (FPI), following the protocol described by Redmond et al. [42]. The FPI will be used as a valid quantitative multiplanar measure of static foot biomechanics [42]. Foot posture will be assessed to classify foot alignment and build on previous research, which has identified a pronated foot type as a risk factor in MTSS development [2]. Furthermore, individuals with a pronated foot type are reported to be almost twice as likely to develop MTSS compared to individuals with a normal or supinated foot type [2].

### Treadmill running protocol

Each participant will run on a treadmill (SportsArt Fitness, Tainan, Taiwan) for 5 min, while wearing the shoes in which they complete most of their weekly training (based on distance), and at a pace equivalent to their most recent 10 km race time. The initial 4 min will be an accommodation period to allow participants to achieve their natural running style. Data characterising the participant’s lower limb biomechanics will then be collected during the final 1 min of running [43]. The biomechanical variables are described below.

#### Running kinematics

Lower limb kinematic data will be collected at 100 Hz using three OptoTRAK Certus motion analysis position sensors (Northern Digital Inc., Ontario, Canada). Prior to each trial infrared emitting diodes will be attached bilaterally to the first, second and fifth metatarsal heads; navicular; posterior calcaneus; medial and lateral malleoli; a rigid body housing three markers at 50% of the anterolateral aspect of the tibial shaft, tibial tuberosity, medial and lateral femoral condyles, a rigid body housing three markers at 50% of the length of the anterolateral thigh, greater trochanter, anterior superior iliac spine, and posterior superior iliac spine so that these sites can be automatically tracked. Double-sided toupee tape (Creative Hair Products, Melbourne, Australia) and 3M transpore plastic tape (Livingstone International Pty Ltd., Rosebery, Australia) will be used to attach the infrared emitting diodes directly to the participant’s skin and shoes. Prior to motion capture, a standing calibration file will be collected [43]. The infrared emitting diodes will then be tracked over an entire gait cycle to enable relevant kinematic variables to be calculated later.

Three-dimensional motion capture will be used to quantify each participant’s step width; foot strike pattern; tibial rotation; hip, knee and ankle joint angle and rearfoot inversion and eversion while they are running. Analysis of kinematic variables will include filtering the raw marker position using a fourth order low pass Butterworth filter with a padding point as described by Willems et al. [44]. Kinematic data will be processed using Visual3D (C-Motion, Germantown, MD) and analysed to measure hip, knee and ankle angle at initial contact; the maximum angle at these joints during stance and the angle at toe off, as well as the maximum and minimum angular velocity at these joints during the gait cycle [45].

#### Muscle activity during running

Neuromuscular activity for the peroneus longus (PL), peroneus brevis (PB), TA, SOL, GM, GL and the extrinsic toe flexors will be quantified during running using a Delsys Trigno™ wireless system (Delsys Inc., Boston, USA) and following the surface EMG (sEMG) for a non-invasive assessment of muscles (SENIAM) guidelines. Neuromuscular activity of the extrinsic toe flexors (flexor hallucis/digitorum longus) will be collected following the protocol described by Peter et al. [46]. Surface EMG signals will be collected bilaterally (2000 Hz) with the participant’s skin prepared following standard guidelines [47]. Electrode placement will be achieved by palpating each muscle during manually-resisted contractions to outline the muscle belly and ensure anatomic variability is taken into consideration. Electrodes with an inter-electrode distance of 10 mm will be placed along the length of the muscle with electrode bars perpendicular to the muscle fibre direction.

Raw sEMG files will first be visually inspected to discard trials contaminated with noise or movement artefact. The raw sEMG signals will then be filtered using a zero-phase-shift, fourth order high pass Butterworth filter and full wave rectified using a low pass Butterworth filter to obtain liner envelopes (mV) [48]. Once filtered, muscle burst onsets and duration will be determined as a burst exceeding 3 standard deviations above a baseline value for a minimum of 100 ms and receding below 3 standard deviations for muscle burst offsets [49]. Trace signals will then be manually adjusted based on visual inspection [49]. Muscular power will be determined using a root mean square (RMS) calculation of the sEMG amplitude. Filtered data will be mathematically squared, then calculated over an interval period determined upon trace visualisation. Surface EMG amplitudes collected during the running protocol will be normalised using the highest mean response for each muscle [50].

#### Plantar pressure distribution

Each participant’s plantar pressure distribution during the running protocol will be measured (100 Hz) using Pedar-X (Novel_gmbh_, Munich, Germany) insoles. Before data collection, the insoles will be calibrated according to the manufacturer’s instructions (Novel_gmbh_, Munich, Germany). The insoles (150 mm × 100 mm × 40 mm, 400 g; 99 sensors) will be placed inside the participant’s shoe and attached to the Pedar-X box, which will be secured to the participant using a running vest. Before each trial, each insole will be zeroed following standard procedures (Novel_gmbh_, Munich, Germany). From the raw data, peak pressure (kPa), peak force (N), contact area (cm^2^), pressure-time integral (kPa.s) and force-time integral (N.s) will be derived for specific locations of the foot, namely the rearfoot (30% of the foot length), midfoot (30% of the foot length) and forefoot (40% of the foot length) [51].

Means and standard deviations for the previously listed variables for running kinematics, muscle activity and plantar pressure distribution will be calculated and averaged over 10 gait cycles for each participant.

### Lower limb endurance

**Fig. 2:**
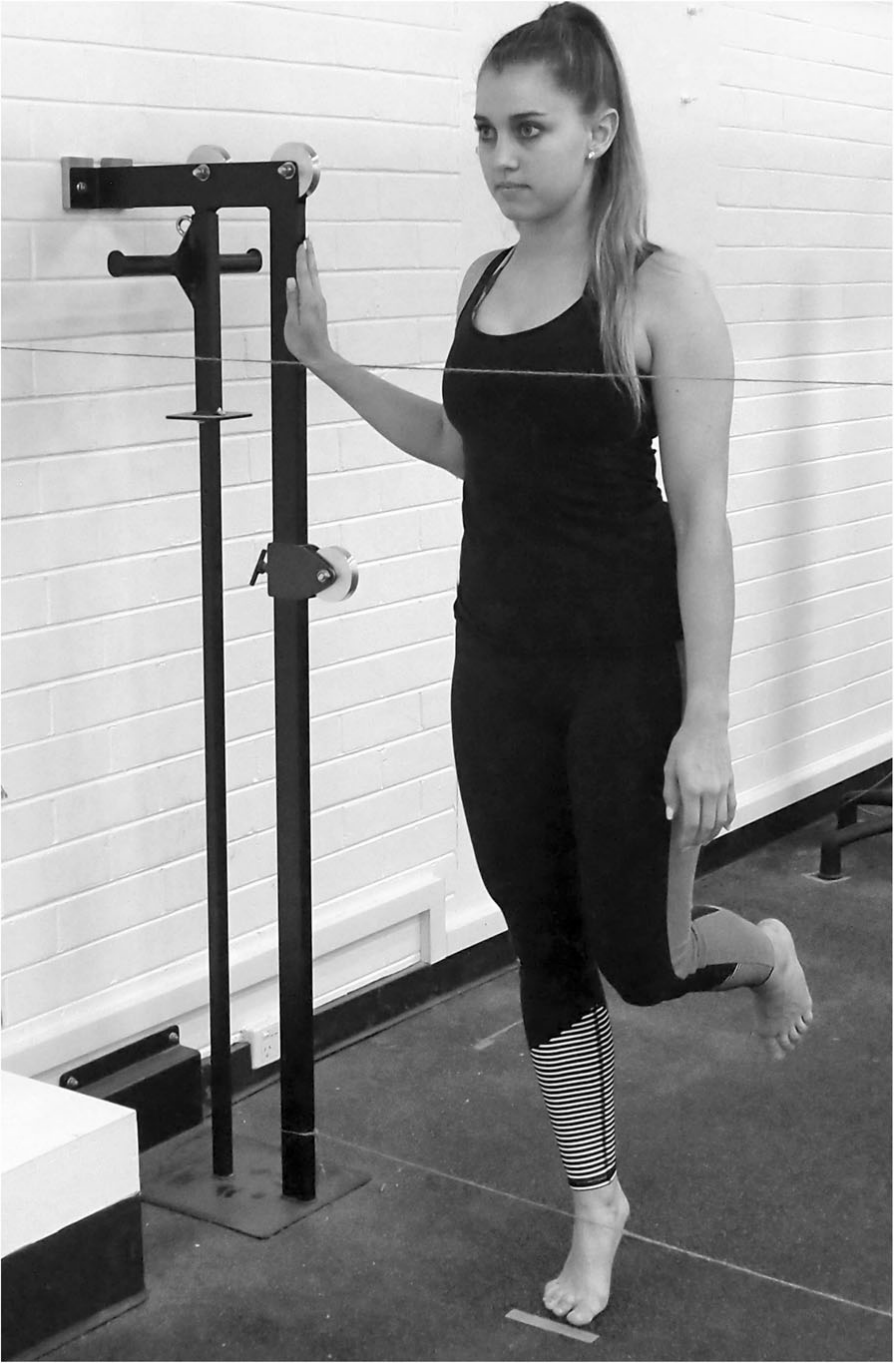
Participant set up for heel raise endurance test

Lower limb muscle endurance will be assessed bilaterally using a heel raise test described by Ross et al. [52]. Following familiarisation with the protocol, each participant will attempt to perform as many single leg heel raises as possible. In brief, one piece of string will be positioned horizontally, approximately 2 cm anterior to the participant’s pectoral muscles. A second piece of string will be placed horizontally between two uprights, which will be adjusted by the chief investigator so that the string contacts the proximal dorsal aspect of the foot while the participant is in maximal ankle plantar flexion, as shown in Fig. 2. The test will be terminated if a participant leans forward and touches the piece of string positioned at the level of their pectorals three times, if they flex the ipsilateral knee, if the dorsal aspect of the foot does not contact the lower string for three consecutive repetitions, or if the participant can no longer continue. Heel raise endurance will be reported as the maximum number of repetitions a participant can achieve.

### Prospective injury and training monitoring

After baseline testing, participants will be tracked for the next 12 months to document all weight bearing activity using a choice of electronic or paper monthly diary entries. They will record: training distance (km), training type (long run, easy run, tempo/race pace run, interval/speed run, walk, strength training, sports match), training duration (minutes), races completed, race distance, race type (trail, road, triathlon, Iron Man), changes in footwear, illness, injuries and subsequent time off running. An injury will be defined as a musculoskeletal impairment for which a participant takes more than seven days off weight bearing activity or seeks treatment from a health professional. For any injury, participants will be required to complete a Sports Medicine Australia Sports Injury Tracker Report [53]. Data will be collated monthly and followed up if not returned. In the case of any injury, participants will be required to immediately notify the chief investigator, who will clarify details of the injury. Upon suspicion of MTSS, participants will be required to attend a follow up session at the Biomechanics Research Laboratory to confirm a diagnosis of MTSS. MTSS will be defined as diffuse pain induced by exercise along the posteromedial tibial border that is non-inclusive of pain from ischaemic origin or stress fracture [2]. Participants, who develop lower limb injuries other than MTSS, will be excluded from analysis if their injury requires complete rest for a period of greater than seven days.

### Statistical analysis

Following the 12-month follow-up period, participants will be divided into two groups: those who developed MTSS (MTSS injured group) and those who did not develop MTSS (uninjured group). Means and standard deviations for each dependent variable for the two groups will be calculated and reported. Data will then be subject to tests of normality using the Kolmogorov-Smirnov test. To identify the risk factors associated with the development of MTSS, logistic regression analysis will be used to analyse the data. Odds ratios (OR) and 95% CI will be calculated. Data will be analysed using SPSS software (Version 23, SPSS Inc., IBM, Armonk, NY, USA) with a significance level being established at *p* ≤ 0.05 to limit a type 1 error to 5%.

## Discussion

This prospective study is being conducted to determine characteristics associated with the development of MTSS in distance runners. The debilitating effects of MTSS can cause individuals to take substantial time off running. For example, a randomized controlled trial reported that runners in three treatment groups took an average of 102–118 (SD 52–64) days to recover sufficiently to complete an 18-min run [54]. This length of time away from running is unsatisfactory for most long-distance runners and can potentially end sporting careers [5]. Thus, further prospective research is needed to systematically determine what characteristics are associated with long distance runners developing MTSS.

In addition to factors discussed in the introduction to this paper, the application of findings from previous research is also limited to younger runners because these studies have assessed runners aged 15–26 years [3, 13, 19]. However, the average age of Australian marathon participants is 36 and 38 years for females and males, respectively [55]. Therefore, research is needed to assess factors associated with the development of MTSS in participants who are more representative of the distance running population.

Although numerous studies have assessed gait parameters of individuals with and without MTSS, these studies also have limitations [56–61]. For example, Willems’ et al. [59] prospective study identified risk factors associated with exercise related lower leg pain in physical education students. Willems et al. [59] reported that individuals who developed exercise related lower leg pain had a more central heel strike, increased pronation and more lateral roll off compared to controls. Exercise related lower leg pain, however, includes compartment syndrome, periostitis, stress fracture, as well as MTSS. Subsequently, future prospective studies are required to determine whether these findings are consistent in participants with MTSS alone. Numerous cross-sectional studies have also assessed static and dynamic lower limb measures in MTSS and asymptomatic populations [56–58, 60, 61]. Consensus exists between studies, concluding that MTSS symptomatic individuals display a more pronated foot during standing and gait compared to asymptomatic controls [56–58, 60, 61]. However, we are unable to determine whether findings from these studies are a cause or effect of MTSS. Therefore, our prospective study will aim to address these limitations.

Distinguishing features of this study are the prospective assessment of lower leg muscle thickness and CSA using B-mode ultrasound and three-dimensional running gait analysis of distance runners. To date, this is the first study to explore individual lower leg muscle thickness and CSA in relation to the development of MTSS. By assessing the composition of lower leg musculature and its contribution to changes in overall lower leg girth this study will aim to determine whether reduced lower leg girth is a risk factor in MTSS development in distance runners and, if so, which specific muscles contribute to this reduction in lower leg girth. This study will also determine whether an imbalance among muscles in the lower leg contribute to the development of MTSS. This would build on previous work by Yuksel et al. [62] who reported that MTSS may be caused by a strength imbalance between the invertor and evertor muscles of the foot in favour of the evertor muscles.

We acknowledge there are some limitations to our study. Firstly, due to constraints associated with the ultrasound imaging probe we are unable to image the CSA of all lower leg muscles. This is because the CSA of soleus and gastrocnemius exceed the width of the probe and it is not feasible to gain access to a wide enough probe. Secondly, the tibialis posterior is unable to be imaged due to its depth in the lower leg exceeding the maximum depth of the probe. Data will be collected for the lower leg muscles; TA, P, FDL, FHL, GM, GL and SOL to gain a global view of lower leg musculature and muscles previously implicated in MTSS development [24, 62, 63]. Findings of this study will be most applicable to distance runners because the participants will be recruited from this sector of the population. Distance runners have been used in this study to avoid numerous confounding variables associated with athletes from multi-sports, while maximising the likelihood of repetitive loading exposure. It is possible that biomechanical profiles obtained during treadmill running may not entirely reflect those obtained during overground running. However, evaluation of kinematic and muscular activation profiles that closely resemble those experienced during distance running necessitates treadmill use.

We have therefore reported our methodology for a prospective study that is investigating characteristics associated with the development of MTSS in distance runners.

The study described in this paper will aim to evaluate lower limb muscle morphology and function of distance runners who do and do not develop MTSS in order to identify modifiable characteristics that can be targeted in future studies to better manage the injury or, preferably, prevent individuals developing MTSS.

## Abbreviations

CSA: Cross sectional area
FDL: Flexor digitorum longus
FHL: Flexor hallucis longus
FPI: Foot posture index
GL: Gastrocnemius lateral head
GM: Gastrocnemius medial head
MTSS: Medial tibial stress syndrome
MVC: Maximal voluntary contraction
P: Peroneals
PB: Peroneus brevis
PL: Peroneus longus
ROM: Range of motion
sEMG: Surface electromyography
SOL: Soleus
TA: Tibialis anterior
